# Serial mediation effects of reflective practice and creative expression on artistic skills development: a cross-cultural analysis in university art education

**DOI:** 10.3389/fpsyg.2025.1528241

**Published:** 2025-06-20

**Authors:** Mengdie Li, Alekseeva Galina Vasilyevna

**Affiliations:** ^1^Academy of Fine Arts, Shanxi Normal University, Taiyuan, China; ^2^Academy of Culture and Arts, Russian Far Eastern Federal University, Vladivostok, Russia

**Keywords:** cultural exposure, reflective practice, creative expression, artistic skills development, experiential learning theory

## Abstract

**Introduction:**

Numerous research efforts have explored the antecedents of artistic skills development among painting students. However, the role of cultural exposure in fostering this development has been largely overlooked. The study aims to address this gap by examining cultural exposure as a prelude to artistic skills development. The study also explores the intermediary pathways through which cultural exposure might stimulate artistic skills development.

**Methods:**

We employed the experiential learning theory to conceptualize the proposed model and collected data from 300 painting majors using a stratified random sampling technique to ensure cultural diversity. Using the Structural Equation Modeling technique, we analyzed the measurement and structural models.

**Results:**

Results indicate that cultural exposure is significantly linked to artistic skills development. Furthermore, reflective practice and creative expression significantly mediate this phenomenon. The study also finds significant serial mediation effects of cultural exposure on reflective practice and then creative expression, leading to enhanced artistic skills development.

**Discussion:**

Our findings offer substantial policy implications that emphasize the need for curricula to encapsulate both cultural immersion and self-reflection practices as a mean to foster artistic skills development in university art education.

## Introduction

Despite substantial research on artistic skills development, the determining factors of artistic skills development still lack a clear consensus. Previous studies suggest that university students’ artistic performance is influenced by various factors, such as cognitive and developmental abilities (Chen, 2020; Pesout and Nietfeld, 2021), level of academic achievement and economic background (Breinholt and Jæger, 2020), religious beliefs (Cusack, 2024), political system (Boorsma and Chiaravalloti, 2024), and culture (Auslander, 2022). In China, the economy has surged over the past 20 years (Sullivan, 2023); nonetheless, a disparity in resources and cultures persists between urban and rural regions. Therefore, it is essential to comprehend the development of creativity in students from a comparative viewpoint. Moreover, recent years have seen a significant increase in the number of foreign students studying in China, offering a valuable opportunity to assess cross-cultural perspectives in university art education.

Of particular interest is the influence of the length of cultural exposure on students’ artistic performance. For instance, Wong and Vong (2023) conducted a study among children aged 5 to 9 years and found that older children performed better on innovation tasks as compared to younger ones. Such findings suggest that cultural exposure plays a crucial role in stimulating people’s perception of creativity and innovation. Similarly, insights from another cross-cultural comparison study indicated that creativity is more closely associated with exposure to multiple cultures (Maddux et al., 2021). However, the relationship between cultural exposure and artistic skills development remains unexplored and warrants further investigation. By investigating the direct relationship between cultural exposure and artistic skills development, the study aims to fill the gap in understanding how diverse cultural experiences enhance artistic skills development among university art students.

Recent research emphasizes that the impact of cultural exposure on artistic performance cannot be fully understood without considering key psychological processes that facilitate learning and creativity (Kitayama and Salvador, 2024). In line with this, we build on the Kolb’s experiential learning theory (Kolb et al., 2014) to introduce reflective practice and creative expression as the mediating mechanisms that facilitate the culmination of cultural exposure in enhanced artistic skills development. According to the experiential learning theory (Kolb and Kolb, 2009), learning occurs through a cyclic process where individuals engage in concrete experiences (i.e., cultural exposure), reflect on these experiences (i.e., reflective practice), and then use these insights to gain new knowledge and skills through active experimentation (i.e., creative expression). The study aims to integrate cultural facets into individual’s learning experiences, stimulating cognitive and emotional process that allows individuals to generate and manifest new and original ideas, especially in artistic contexts. The interplay between these cultural and psychological factors brings about a deeper understanding of how diverse cultural experiences influence the development of artistic skills. In light of the above, the study attempts to contribute to this limited stream of research and fill the gap from a cross-cultural perspective. The study develops a cultural exposure framework, investigating how diverse cultural experiences influence the artistic skills development of university art students. Specifically, the study aims to test (1) the direct influence of cultural exposure on university art students’ artistic skills development; (2) the indirect effect of cultural exposure on artistic skills development through reflective practice and creative expression; (3) the serial mediation effects of reflective practice and creative expression between cultural exposure and artistic skills development.

In light of the above, this study contributes to the limited stream of research by developing a cultural exposure framework and investigating how diverse cultural experiences influence the artistic skills development of university art students. Based on this rationale, the study aims to achieve the following objectives:

1. To examine the direct influence of cultural exposure on university art students’ artistic skills development.
2. To investigate the indirect effects of cultural exposure on artistic skills development through reflective practice and creative expression.
3. To assess the serial mediation effects of reflective practice and creative expression between cultural exposure and artistic skills development.

## Literature review

### Experiential learning theory

Experiential learning theory (ELT), proposed by Kolb (1984), refers to “the process by which knowledge is generated through the transformation of experience. Knowledge arises from the synthesis of understanding and altering experience” (p. 41). The ELT encompasses a four-stage model, with two dialectically related models of grasping knowledge, such as concrete experience and abstract conceptualization; and two dialectically related models of transforming experience, such as reflective observation and active experimentation (Kolb et al., 2014). Over the years, the theory has rich theoretically underpinning with its applications in various domains, such as education (Kolb and Kolb, 2017), business, psychology (Bell and Bell, 2020; Kolb et al., 2014), and healthcare (Irwin et al., 2024; Johnson, 2020). In the present study, we anticipate that ELT provides theoretical foundation to associate cultural exposure (i.e., concrete experience) with artistic skills development (i.e., active experimentation) through the mediating roles of reflective practice (i.e., reflective observation) and creative expression (i.e., abstract conceptualization). The subsequent section presents hypothetical inferences of the proposed model embedded in the ELT.

### Cultural exposure and artistic skills development

The study posits that cultural exposure plays a pivotal role in shaping the creative and artistic abilities of individuals as they are exposed to different cultural experiences that nurture inspiration and learning. Cultural exposure is defined as experiences associated with a region that facilitate the development of familiarity or comprehension of the norms, values, and beliefs of that region (Anderson, 2022). In this study, we adopt a constructivist and experiential viewpoint to frame culture. From this perspective, culture is not treated as a static set of attributes but as a dynamic, interactive process in which individuals derive meaning through exposure, interpretation, and participation in culturally embedded practices (Cerulo et al., 2021). This aligns with experiential learning theory (Jonathan and Laik, 2021), which emphasizes learning through lived experiences and reflection. Hence, cultural exposure is conceptualized not merely as geographic relocation or surface-level interaction but as an immersive process that shapes cognition, self-awareness, and creative potential. It is widely agreed that an individual’s experiences with exposure to another culture affects their cultural exposure (Dias et al., 2020; Tan et al., 2015). However, in a foreign environment, avoiding engagement with the local culture would likely result in a minimum cultural experience. The degree of exposure is characterized by the types of experiences overseas, including participation in cultural activities, such as engaging with the local culture. This essentially involves assessing the degree of exposure to the culture (Wong and Vong, 2023). For instance, an individual who frequently purchases lunch at a local café or engages with residents would certainly have a more profound comprehension of the local culture than someone who does not.

Researchers have discovered evidence supporting the notion that the most efficacious approach to learning about a new culture is through ‘concrete experience,’ which entails immersion in an experience while utilizing emotional engagement and comprehension (Ramirez, 2023). Consequently, an individual who engages with a culture, including interactions with residents, is likely to get greater insights from their cultural exposure than someone who does not. In this perspective, studies suggest that experiential learning is crucial for developing the behavioral patterns vital for cultural intelligence (Pasztor, 2021). In addition, research suggests that when students interact with various cultural experiences, it not only helps them in facilitating their technical skills but also improves cognitive flexibility (Bernardo and Presbitero, 2018), which is essential for artistic innovation. Similarly, insights drawn from others studies corroborate that individuals gaining multicultural experiences are more likely to experience enhanced creativity by broadening cognitive frameworks (Cerulo et al., 2021), which allow individuals to generate more novel and diverse ideas.

### Reflective practice and creative expression

We further anticipate that cultural exposure plays a crucial role in fostering reflective practice, particularly in creative fields like art and design. Reflective practice is “the ability to reflect on one’s actions so as to take a critical stance or attitude towards one’s own practice and that of one’s peers, engaging in a process of continuous adaptation and learning” (Donald, 1983; Schön, 2017). According to (Kolb et al., 2014), reflective practice encompasses self-assessment and deep thinking, which allows individuals to reflect upon new cultural insights and incorporate them into their work. Both of these elements of reflective practice are closely tied to cultural intelligence (Pasztor, 2021), which refers to “the skill to relate and work effectively in culturally diverse situations.” According to the ELT (Kolb, 1984), reflective practice involves the transformation of concrete experience–i.e., cultural exposure–into meaningful knowledge. When individuals are exposed to different cultural experiences, they critically assess these cultural artefacts and reflect on how these experiences influence their learning and creative behavior (Ramirez, 2023).

Kolb contends that according to the ELT, reflection entails the “internal transformation of experience,” necessitating reflective “cognitive complexity and the capacity for critical thinking” (Kolb and Kolb, 2009). The author further asserts that experiential learning environments, particularly those that incorporate diverse learning elements, facilitate a distinct model of education compared to conventional classrooms by providing students the chance to participate in more profound and culturally significant learning experiences. Moreover, Kolb (1984) elucidates that experience learning can lead to enhanced retention as the student transitions between various experiential learning modes within the learning cycle. Consequently, from the perspective of experiential learning, fostering and augmenting students’ artistic skills development necessitates the exploration of concepts and reflection on the learning process, alongside deriving insights from diverse cultural experiences and adapting perspectives to generate new knowledge and artistic expression.

The theoretical underpinnings of this relationship can also be understood from the perspective of the acculturation complexity model (van der Zee and van Oudenhoven, 2022). Acculturation is “a process of social, psychological, and cultural transformation resulting from the integration of two cultures while adjusting to the dominant culture of society” (Tanenbaum et al., 2020). The acculturation complexity model (van der Zee and van Oudenhoven, 2022) leverages individuals to adapt cultural strategies and acknowledge cognitive complexity, thereby expanding the breadth and depth of their cognitive flexibility. For instance, Chinese-American adults who assimilated both cultures articulated their understanding of Chinese and American cultures in more nuanced terms compared to monoculturals who prioritized a single culture. The cognitive advantage of biculturals who equally embrace both cultures appear to extend beyond culture-specific tasks to encompass creativity.

Subsequently, we posit that cultural exposure is linked with creative expression through the transformation of reflective experience in an innovative and novel manner. “Creativity” is characterized as the generation of ideas that are both innovative and practical (Tan et al., 2019), and various studies indicate that creativity consistently enhances when individuals encounter intercultural concepts, individuals, or settings. The initial empirical study regarding this possibility was the creation of the Multicultural Experience Survey (Aytug et al., 2018), which assesses various dimensions of multicultural experiences, such as duration spent abroad, overall exposure to foreign cultures, number of foreign languages spoken, parents’ countries of origin, and the countries of origin of participants’ five preferred cuisines, friends, and musicians. Numerous research has demonstrated that persons who get elevated scores on this measure (or a comparable scale) display more creativity (Leung et al., 2008). Moreover, studies indicate that even a short duration of exposure to contrasting cultural aspects might augment creativity. For instance, viewing consecutive slideshows that juxtapose Chinese and American cultures can enhance participants’ creativity compared to those viewing slideshows focused solely on one culture (Chun, 2000). Additional studies have concentrated on particular varieties of multicultural encounters. Numerous studies indicate that creativity is enhanced when individuals reside abroad (Zhao et al., 2019), engage in work abroad (Ali et al., 2019), and, in certain instances, pursue studies abroad (Durdas et al., 2022) or travel internationally (Rátz, 2017). Leung et al. (2008) discovered that the duration MBA students resided outside their home country was a predictor of creativity across various tasks measuring insight, divergent thinking, and convergent thinking, with this effect mediated by the degree of cultural adaptation participants experienced in the host country. Subsequent research by Cho and Morris (2015) indicated that profound understanding of the new culture significantly contributed to heightened creativity after experiences of living abroad. Moreover, longitudinal research subsequently identified enhancements in creativity among expatriate workers after periods of employment overseas (Kim, 2020). The impact of residing abroad also influences institutional inventiveness. Similarly, in the global fashion industry, Thomas (2020) discovered that the duration creative directors—who are the effective leaders of premier fashion houses—spent working internationally correlated positively with the creativity ratings of their companies’ fashion collections by industry experts.

In a nutshell, an extensive review of literature highlights the critical role that cultural exposure plays in shaping university art students’ artistic skills development through the intermediary roles of psychological processes that mediate this pathway. Based on the ELT, we anticipate that cultural exposure serves as a concrete experience that facilitates learning through reflective practice, which further translates into creative expression. Subsequently, individuals observing and adapting to different cultural experiences tend to expand their cognitive frameworks, which allows them to generate novel ideas and enhance their artistic skills. Hence, the study hypothesizes that the cognitive and creative aspects of artistic growth can be stimulated through cultural exposure, which facilitates reflective practice and encourage creative expression.

**Figure 1:**
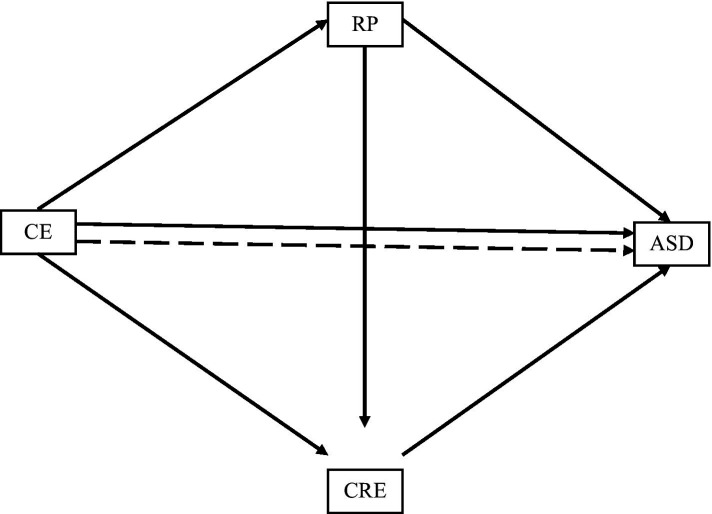
Conceptual model (CE stands for cultural exposure; RP stands for reflective practice; CRE stands for creative expression; ASD stands for artistic skills development; straight lines show direct effects; dotted line reflects indirect effect).

Based on these aforementioned arguments, the study puts forth the following research hypotheses (see Figure 1):

*H1*: Cultural exposure will have a significant positive impact on artistic skills development.

*H2*: Cultural exposure will have a significant positive impact on reflective practice.

*H3.* Reflective practice will have a significant positive impact on creative expression.

*H4*: Reflective practice will mediate the relationship between cultural exposure and creative expression.

*H5*: Creative expression will mediate the relationship between reflective practice and artistic skills development.

*H6*: Cultural exposure and artistic skills developments’ direct relationship will be serially mediated by reflective practice and creative expression.

### Research methods

We employed a quantitative survey-based technique to investigate the impact of cultural exposure on artistic skills development among university art students, specifically painting majors. For this reason, a cross-sectional research design was chosen to collect data from the key respondents at a particular time. Data collection was carried out over a period of 4 months, from March to June 2024. The study exclusively targeted students majoring in painting who were enrolled in university-level art education programs. While the term “art education” is often used broadly to encompass multiple disciplines, our focus on painting majors was deliberate to maintain conceptual specificity and ensure coherence with the experiential learning processes central to the study.

Participation of the key respondents was voluntary and an informed consent was taken prior to their participation in the study. The cover letter, distributed along-side questionnaires, contained such information. In addition, aligning with our study’s objectives, we ensured that participants of this study should be exposed to cultural exchange activities, such as cross-cultural art programs, international collaboration, etc. Moreover, in order to assure cultural representation, we included participants from diverse cultural backgrounds. For this reasons, multiple universities were shortlisted, and domestic and international candidates were involved in this study.

A stratified random sampling technique was employed to ensure cultural diversity in the sample. The stratification was based on students’ self-identified cultural backgrounds—specifically Western (e.g., Europe, North America), Eastern (e.g., East and South Asia), African, and Latin American regions. Within each cultural stratum, participants were randomly selected from the enrolled painting majors to participate in the study. This approach enabled proportionate representation across cultural groups while preserving the randomness of participant selection within each category, thus strengthening the generalizability of the findings to culturally diverse student populations in university art education.

We distributed questionnaires to 370 students and received 323 completed forms. Of these, 23 questionnaires were excluded from the final analysis due to substantial missing data or response patterns indicating low engagement (e.g., identical answers across all items), which could compromise the reliability of the findings. Thus, 300 valid responses were retained for further analysis using multivariate data techniques.

The demographic breakdown of the sample reveals a fairly balanced representation of gender, with 53% female and 47% male students. In terms of age distribution, the majority of participants (42%) fall within the 21–23 age range, followed by 33% aged 18–20, 18% aged 24–26, and a smaller portion (7%) between 27 and 30 years old (Table 1). Cultural background is notably diverse, with 55% of students coming from Eastern (East and South Asia) regions, 12% from Western (Europe and North America) areas, 8% from Latin American backgrounds, and 10% from African origins. The academic year distribution is relatively even, with second-year students making up 28%, followed by third-year students at 26%, fourth-year students at 24%, and first-year students at 22%. The sample also shows a balance in international student status, with 52% being domestic students and 48% being international students, providing a culturally varied cohort for the study.

**Table 1:** Participants demographics.

Demographic	Percentage	Number of students
Gender		
Female	53%	159
Male	47%	141
Age		
18-20 years	33%	99
21-23 years	42%	126
24-26 years	18%	54
27-30 years	7%	21
Cultural Background		
Western (Europe, North America)	12%	36
Eastern (East and South Asia)	55%	165
African	10%	30
Latin American	8%	24
Academic Year		
First-year students	22%	66
Second-year students	28%	84
Third-year students	26%	78
Fourth-year students	24%	72
International Student Status		
Domestic Students	52%	156
International Students	48%	144

### Research instruments

Research instruments for this study have been adapted from previous studies with well-established reliability to ensure their appropriateness with the present study. These questionnaires were slightly modified to capture the art education perspective in this study. To ensure contextual relevance, minor modifications were made to the original instruments to align the wording with the domain of university-level art education, specifically painting majors. These modifications involved rephrasing general references (e.g., “work tasks” or “performance”) to discipline-specific terms such as “artistic tasks” or “creative output.” The core constructs and original item structures were preserved to maintain content validity. To assess the impact of these changes on reliability, we conducted a pilot test with 30 participants from a similar demographic. The results showed Cronbach’s alpha values above the 0.75 threshold for all scales, confirming acceptable internal consistency following modification. Hence, the adapted instruments were considered reliable for full-scale data collection.

Moreover, we used two versions of questionnaires in order to adhere to cross-cultural perspectives of the study. We used back-translation technique, suggested by Brislin (1970) to develop the questionnaire in Chinese, as this is a medium of instructions in educational institutions across China. Besides, participants were also provided English version of the questionnaire in order to maximize response accuracy and accommodate participants who may be comfortable with English. Through this dual-language approach, we ensured that language barrier did not interfere with the comprehension of the items. Further, a five-point Likert scale questionnaire technique was chosen in this study with items measure complete dissatisfaction as 1 and complete satisfaction as 5. For measuring cultural exposure, we adapted the scale items from the Metacognitive Cultural Intelligence (CQ) scale (Ang et al., 2007), consisting of four items. Similarly, we modified the reflection and critical reflection scale developed by Kember et al. (2000), consisting of five items. For measuring creative expression, we adapted the scale items from the Creative Self-efficacy scale (Tierney and Farmer, 2002), consisting of four items. Besides, we modified the creativity and skill development scale developed by Amabile (1983), consisting of five items.

## Results

The analytical technique used in this study involves the assessment of the proposed model using SmartPLS software (Ringle et al., 2012). As the main purpose of this study is prediction of the proposed model, (Hair et al., 2020) endorsed that PLS SEM is a more appropriate technique. In addition, Shmueli et al. (2016) reinforced that “PLS-SEM primarily focuses on the interplay between prediction and theory testing and results should be validated accordingly.” Thus, in accord with the guidelines offered by Hair et al. (2020) about the use of PLS SEM, we tested (1) inter-item reliability, convergent and discriminant validity using the measurement model; and (2) hypotheses testing and predictive capability assessment using the structural model.

### Measurement model

Initially, we conducted Harman’s single factor test utilizing SPSS 24 software to assess the presence of common method bias (Podsakoff et al., 2003). The findings indicated that the first component accounted for 29.10% of the total variance, well below the 40% threshold. Secondly, we utilized a correlation matrix approach, revealing that the maximum inter-construct correlation was 0.813, which remained below the 0.90 cut-off value (Chin et al., 2012). Third, we conducted a comprehensive collinearity assessment utilizing SmartPLS software, revealing that the maximum pathological VIF for all components was 2.112, well below the advised threshold of 3.3 (Hair et al., 2020). Consequently, the studies indicate that common technique bias was not a concern.

The results indicate strong internal consistency and reliability for the constructs measured in the study, as evidenced by the high Cronbach’s alpha values (above 0.75 for all variables), composite reliability (rho_c), and average variance extracted (AVE). All values exceed the common threshold, suggesting that the items for each construct consistently measure their respective concepts. The AVE values, ranging from 0.546 to 0.670, suggest good convergent validity, as each construct explains a substantial portion of the variance in its items (see Table 2).

**Table 2:** Cronbach’s alpha, composite reliability, and AVE.

Variables	CA	rho_a	rho_c	AVE
Artistic skills development	0.829	0.833	0.886	0.661
Creative expression	0.837	0.848	0.890	0.670
Cultural exposure	0.754	0.750	0.843	0.574
Reflective practice	0.785	0.816	0.854	0.546

The Fornell-Larcker and Heterotrait-Monotrait ratios confirm discriminant validity, as each construct shows stronger correlations within its items than with other constructs. The cross-loadings further reinforce this, with each item loading more highly on its respective construct compared to others. For example, the artistic skills development items show strong loadings on their own construct (above 0.76), while their loadings on other constructs are substantially lower. Similarly, creative expression and reflective practice items load primarily on their respective factors, indicating distinct and well-separated constructs. Overall, these results suggest a robust measurement model with good reliability and validity across the studied variables (See Tables 3–5).

**Table 3:** Fornell-Larcker.

Variables	Artistic skills development	Creative expression	Cultural exposure	Reflective practice
Artistic skills development	0.813			
Creative expression	0.636	0.819		
Cultural exposure	0.655	0.502	0.758	
Reflective practice	0.651	0.512	0.424	0.739

**Table 4:** Heterotrait-monotrait.

Variables	Artistic skills development	Creative expression	Cultural exposure	Reflective practice
Artistic skills development				
Creative expression	0.745			
Cultural exposure	0.812	0.586		
Reflective practice	0.800	0.619	0.513	

**Table 5:** Cross loadings.

Indicators	Artistic skills development	Creative expression	Cultural exposure	Reflective practice
ASD1	0.761	0.421	0.638	0.441
ASD3	0.844	0.609	0.578	0.575
ASD4	0.813	0.565	0.483	0.547
ASD5	0.832	0.455	0.420	0.550
CE1	0.511	0.558	0.675	0.373
CE2	0.449	0.319	0.793	0.248
CE3	0.496	0.242	0.782	0.238
CE4	0.500	0.322	0.775	0.379
CRE1	0.482	0.821	0.324	0.460
CRE2	0.452	0.827	0.370	0.357
CRE3	0.629	0.841	0.508	0.463
CRE4	0.490	0.785	0.414	0.383
RP1	0.368	0.242	0.143	0.516
RP2	0.460	0.445	0.296	0.739
RP3	0.515	0.361	0.353	0.788
RP4	0.433	0.377	0.283	0.756
RP5	0.597	0.436	0.427	0.851

Cross Loadings, the shaded values (or highest values in each row) represent the loading of each item on its respective (intended) construct, indicating how strongly that item correlates with the latent variable it was designed to measure.

### Structural model

**Figure 2:**
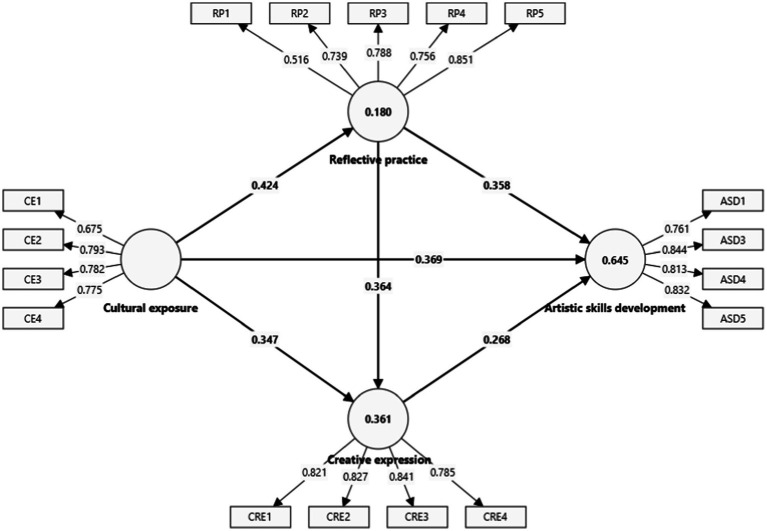
Structural model of cultural exposure and artistic skills development.

The structural path analysis (Table 6 and Figure 2) reveals significant relationships across all hypothesized paths. Cultural exposure has a strong positive direct effect on artistic skills development (H1, *β* = 0.369, *p* < 0.001), with confidence intervals showing this effect is robust (95% CI: 0.247, 0.504). Additionally, cultural exposure significantly predicts reflective practice (H2, β = 0.424, *p* < 0.001), reinforcing its role in encouraging reflective thought processes in students (95% CI: 0.300, 0.548).

**Table 6:** Structural paths and confidence intervals.

Hypotheses	Path	*p*	Confidence intervals	
			2.5%	97.5%
H1: Cultural exposure - > Artistic skills development	0.369	0.000	0.247	0.504
H2: Cultural exposure - > Reflective practice	0.424	0.000	0.300	0.548
H3: Reflective practice - > Creative expression	0.364	0.000	0.205	0.510
H4: Cultural exposure - > Reflective practice - > Creative expression	0.154	0.000	0.080	0.237
H5: Reflective practice - > Creative expression - > Artistic skills development	0.098	0.003	0.040	0.171
H6: Cultural exposure - > Reflective practice - > Creative expression - > Artistic skills development	0.041	0.005	0.017	0.073

Reflective practice positively influences creative expression (H3, β = 0.364, *p* < 0.001), showing that students’ reflective engagement with cultural experiences leads to greater creativity (95% CI: 0.205, 0.510). The indirect path from cultural exposure through reflective practice to creative expression (H4, β = 0.154, *p* < 0.001) further demonstrates that reflective practice mediates this relationship (95% CI: 0.080, 0.237).

The serial mediation effects also hold significant, as the path from reflective practice through creative expression to artistic skills development (H5, β = 0.098, *p* = 0.003) shows a significant positive impact (95% CI: 0.040, 0.171). The serial mediation from cultural exposure through reflective practice and creative expression to artistic skills development (H6, β = 0.041, *p* = 0.005) is also significant (95% CI: 0.017, 0.073). These results emphasize the importance of both reflective practice and creative expression in translating cultural exposure into enhanced artistic skills.

## Discussion

The main purpose of this study was to investigate how cultural exposure influences university students’ artistic skills development, with particular attention paid to the psychological factors that influence this process. Despite some attention to how exposure to diverse cultural elements might improve artistic performance (Niu and Sternberg, 2001; Yi et al., 2013), relatively a small sample of studies has assessed why art students’ cultural exposure might aggravate their artistic skills development, let alone examined the critical roles of reflective practice and creative expression therein. To bridge this theoretical omission, we have drawn from the ELT (Kolb, 1984) to illuminate that (1) the likelihood of improved artistic skills development is contingent upon cultural factors that stimulate this phenomenon, and (2) reflective practice and creative expression reinforce the underlying relationship.

This study provides a fresh perspective by demonstrating that reflective practice as a means to cultural assimilation is a pivotal mechanism that explains how students can transform cultural artefacts in enhanced artistic performance. That is to say, exposure to different cultures may enhance individuals’ capacity to discern their internal processes beyond superficial appearances. This is because reflective practice allows individuals to critically analyze various aspects of cultures and integrate in one’s cognitive processing, thereby expanding the horizons of their cognitive frameworks. The relationship between cultural exposure and reflective practice is an iterative process, whereby diversity in the exposure of cultural elements stretches one’s reflective practice, which in turn, promotes cultural self-understanding by eliciting questions about one’s self and different cultures (Sparrow, 2016).

This phenomenon facilitates true engagement with individuals from different cultures through self-awareness, rather than relying on preconceptions and stereotypes about the “other” (Lu and Wan, 2018). This is further supported by Arieli and Sagiv (2018), who contend that multicultural experiences also manifest in problem-solving scenarios, allowing individuals to autonomously take information from diverse cultures and synthesize it in innovative manners, so broadening cognitive categories by assimilating ostensibly unrelated concepts. Hence, the findings of this study are in harmony with previous literature such as an increased exposure to diverse cultures augment reflective practices (Danso, 2018), which in turn, paves the ways for boosting creative expression (Tan et al., 2020).

Of these, we focus on reflective practice and creative expression as key psychological mechanisms mediating the relationship between cultural exposure and artistic skills development for three main reasons (Kolb, 1984; Schön, 2017). First, cultural exposure stimulates reflective thinking, prompting students to critically analyze their artistic work and learn from diverse cultural perspectives. Second, reflective practice enables students to internalize cultural experiences, allowing them to innovate and apply these insights in new artistic creations. Third, both reflective practice and creative expression directly contribute to enhanced artistic skills, making them essential pathways through which cultural exposure fosters creative growth.

Last but not the least, we relied on the ELT (Kolb, 1984) to empirically assess the proposed model encompassing the relationships between cultural exposure, reflective practice, creative expression, and artistic skills development. This if one of the few studies that have utilized ELT in the context of university education programs (Jonathan and Laik, 2021), particularly painting majors. Also, the complex interplay between the cultural and psychological constructs offers unique perspective, which to date, has never been tested earlier. Underpinned by ELT, we found that cultural exposure significantly enhances artistic skills development, with reflective practice and creative expression serving as key mediators. The results provide valuable insights into how students’ engagement with diverse cultural experiences leads to deeper reflective thinking and more innovative artistic outputs, ultimately fostering the development of their artistic skills. These findings not only contribute to the broader literature on art education but also offer practical implications for integrating cross-cultural elements into creative education programs.

## Limitations

Although our study presents unique theoretical and empirical perspectives on the interplay between cultural exposure, reflective practice, creative expression, and artistic skills development, this study is not free from its limitations. First of all, we collected data using a cross-sectional research design. The choice of a cross-sectional design was guided by the study’s purpose to empirically examine the proposed relationships among variables. However, we contend that future studies should employ longitudinal as well as experimental research designs so that a causation can be established among these variables. Second, our study collected data from China. Although collecting data from various cultural backgrounds provides enriching insights to our study. We recommend that future studies should examine cross-cultural data collected from universities in different cultures. This approach would assist in generalizing the findings. Another limitation of this study is the examination of the serial mediation model. Future research should also explore the intervening paths influencing these links.

Additionally, while this study provides empirical insights through a quantitative lens, we acknowledge that the development of artistic skills in culturally diverse contexts involves deeply personal, emotional, and context-specific experiences. These soft and subjective aspects—central to how individuals internalize culture and transform it into creative output—may not be fully captured through structured survey instruments. Given the interpretive and immersive nature of cultural experiences, future research could benefit from ethnographic or qualitative methodologies (e.g., in-depth interviews, participant observation, or narrative analysis) to explore these dynamics in greater depth and nuance.

## Practical implications

The findings of this study offer several important practical implications. By demonstrating that cultural exposure enhances artistic skills development through the serial mediation of reflective practice and creative expression, the study highlights the value of incorporating diverse cultural experiences into art curricula. Art programs can benefit from integrating more cross-cultural elements, such as international collaborations, exposure to global art forms, and cultural exchange programs, to stimulate students’ creativity and technical proficiency.

Furthermore, encouraging reflective practice within art education is critical for fostering deeper self-assessment and personal growth in students. Educators should emphasize reflection not only on artistic techniques but also on how cultural experiences shape their creative processes. Integrating structured reflection exercises in the curriculum can help students develop the cognitive flexibility needed to translate cultural insights into innovative artistic outcomes.

Lastly, the findings support the idea that fostering creative expression through diverse cultural exposure helps students generate novel artistic ideas. This suggests that art educators should create environments where students are encouraged to experiment and apply cultural influences in their work, broadening their creative horizons and enhancing their artistic performance. By implementing these strategies, art institutions can cultivate a more innovative and culturally aware generation of artists.

## Conclusion

With this investigation, we have sought to expand the previous literature on university art students’ artistic skills development by investigating the critical role of cultural exposure, as well as the roles of reflective practice and creative expression in this process. Both reflective practice and creative expression represent crucial reasons translates cultural exposure into escalated artistic skills development. In brief, we hope that this study serves as a catalyst for future research on how universities can manage the integration between culture and psychological factors to stimulate artistic performance and creativity.

## Data Availability

The original contributions presented in the study are included in the article/Supplementary material, further inquiries can be directed to the corresponding author.
