# Supplementary material for: Serial mediation effects of reflective practice and creative expression on artistic skills development: a cross-cultural analysis in university art education
Source: Front Psychol. 2025 Jun 20;16:1528241. doi: 10.3389/fpsyg.2025.1528241 (PMC12226863; doi:10.3389/fpsyg.2025.1528241)
Supplement: Supplementary file 1 [file Data_Sheet_1.pdf]

| RP1 | RP2 | RP3 | RP4 | RP5 | CRE1 | CRE2 | CRE3 | CRE4 |
|---|---|---|---|---|---|---|---|---|
| 4 | 5 | 4 | 5 | 4 | 5 | 5 | 5 | 4 |
| 5 | 5 | 4 | 4 | 4 | 5 | 5 | 5 | 4 |
| 5 | 5 | 4 | 5 | 4 | 4 | 5 | 5 | 5 |
| 4 | 4 | 5 | 5 | 5 | 5 | 5 | 5 | 4 |
| 5 | 4 | 5 | 4 | 4 | 4 | 5 | 4 | 5 |
| 5 | 5 | 4 | 5 | 4 | 4 | 5 | 5 | 5 |
| 4 | 4 | 5 | 4 | 5 | 4 | 4 | 4 | 5 |
| 4 | 5 | 5 | 5 | 5 | 5 | 4 | 4 | 5 |
| 5 | 4 | 4 | 4 | 4 | 5 | 5 | 5 | 5 |
| 4 | 5 | 5 | 5 | 5 | 5 | 5 | 5 | 5 |
| 5 | 5 | 4 | 5 | 4 | 5 | 4 | 4 | 4 |
| 4 | 4 | 4 | 4 | 4 | 4 | 4 | 4 | 4 |
| 5 | 4 | 5 | 4 | 5 | 4 | 4 | 4 | 3 |
| 5 | 4 | 4 | 4 | 4 | 4 | 3 | 4 | 3 |
| 4 | 5 | 4 | 4 | 4 | 4 | 4 | 4 | 4 |
| 4 | 4 | 3 | 4 | 3 | 3 | 4 | 4 | 3 |
| 5 | 4 | 4 | 4 | 4 | 4 | 4 | 4 | 4 |
| 4 | 4 | 3 | 4 | 4 | 4 | 4 | 4 | 4 |
| 4 | 5 | 4 | 5 | 4 | 5 | 5 | 5 | 4 |
| 4 | 5 | 4 | 4 | 4 | 5 | 5 | 5 | 4 |
| 5 | 5 | 4 | 5 | 4 | 4 | 5 | 5 | 5 |
| 5 | 4 | 5 | 5 | 5 | 5 | 5 | 5 | 4 |
| 3 | 4 | 5 | 4 | 4 | 4 | 5 | 4 | 5 |
| 5 | 5 | 4 | 5 | 4 | 4 | 5 | 5 | 5 |
| 4 | 4 | 5 | 4 | 5 | 4 | 4 | 4 | 5 |
| 4 | 5 | 5 | 5 | 5 | 5 | 4 | 4 | 5 |
| 4 | 4 | 4 | 4 | 4 | 5 | 5 | 5 | 5 |
| 5 | 4 | 5 | 4 | 4 | 4 | 5 | 4 | 5 |
| 2 | 5 | 4 | 5 | 4 | 4 | 5 | 5 | 5 |
| 3 | 4 | 5 | 4 | 5 | 4 | 4 | 4 | 5 |
| 2 | 5 | 5 | 5 | 5 | 5 | 4 | 4 | 5 |
| 3 | 4 | 4 | 4 | 4 | 5 | 5 | 5 | 5 |
| 3 | 5 | 5 | 5 | 5 | 5 | 5 | 5 | 5 |
| 5 | 5 | 4 | 5 | 4 | 5 | 4 | 4 | 4 |
| 4 | 5 | 5 | 5 | 4 | 5 | 4 | 5 | 4 |
| 5 | 4 | 4 | 4 | 4 | 3 | 4 | 5 | 4 |
| 5 | 5 | 4 | 4 | 4 | 4 | 5 | 4 | 5 |
| 4 | 4 | 3 | 4 | 3 | 3 | 4 | 4 | 3 |
| 4 | 4 | 5 | 4 | 4 | 4 | 5 | 4 | 5 |
| 4 | 4 | 4 | 4 | 4 | 4 | 4 | 4 | 4 |
| 5 | 4 | 5 | 4 | 5 | 4 | 4 | 4 | 3 |
| 4 | 4 | 4 | 4 | 4 | 5 | 5 | 5 | 5 |
| 4 | 5 | 4 | 5 | 4 | 5 | 5 | 5 | 4 |
| 4 | 5 | 5 | 5 | 4 | 4 | 4 | 4 | 5 |
| 5 | 5 | 5 | 5 | 5 | 5 | 4 | 4 | 4 |
| 5 | 4 | 5 | 4 | 4 | 4 | 5 | 4 | 5 |

| | | | | | | | | |
|---|---|---|---|---|---|---|---|---|
| 5 | 4 | 5 | 5 | 5 | 5 | 5 | 5 | 4 |
| 4 | 4 | 5 | 4 | 4 | 4 | 5 | 4 | 5 |
| 5 | 5 | 4 | 5 | 4 | 4 | 5 | 5 | 5 |
| 4 | 4 | 4 | 4 | 4 | 5 | 5 | 5 | 5 |
| 4 | 4 | 4 | 4 | 4 | 5 | 5 | 5 | 5 |
| 5 | 4 | 4 | 4 | 4 | 5 | 5 | 5 | 5 |
| 4 | 5 | 5 | 5 | 5 | 5 | 5 | 5 | 5 |
| 5 | 5 | 5 | 5 | 4 | 4 | 4 | 4 | 5 |
| 4 | 4 | 4 | 4 | 4 | 5 | 5 | 5 | 5 |
| 5 | 5 | 4 | 5 | 4 | 4 | 5 | 5 | 5 |
| 5 | 5 | 5 | 5 | 4 | 4 | 4 | 4 | 5 |
| 4 | 4 | 4 | 4 | 4 | 5 | 5 | 5 | 5 |
| 4 | 4 | 4 | 4 | 4 | 5 | 5 | 5 | 5 |
| 4 | 5 | 4 | 5 | 4 | 4 | 5 | 5 | 5 |
| 5 | 4 | 4 | 4 | 4 | 5 | 5 | 5 | 5 |
| 4 | 5 | 5 | 5 | 4 | 4 | 4 | 4 | 5 |
| 5 | 4 | 5 | 4 | 4 | 4 | 5 | 4 | 5 |
| 4 | 5 | 4 | 5 | 4 | 4 | 5 | 5 | 5 |
| 5 | 5 | 4 | 5 | 4 | 4 | 5 | 5 | 5 |
| 5 | 4 | 5 | 4 | 4 | 4 | 5 | 4 | 5 |
| 4 | 4 | 4 | 4 | 4 | 4 | 3 | 4 | 3 |
| 4 | 5 | 4 | 4 | 4 | 4 | 4 | 4 | 4 |
| 4 | 4 | 3 | 4 | 3 | 3 | 4 | 4 | 3 |
| 5 | 4 | 4 | 4 | 4 | 4 | 4 | 4 | 4 |
| 4 | 4 | 3 | 4 | 4 | 4 | 4 | 4 | 4 |
| 5 | 5 | 4 | 5 | 4 | 5 | 5 | 5 | 4 |
| 5 | 5 | 5 | 5 | 5 | 1 | 1 | 5 | 4 |
| 4 | 3 | 4 | 4 | 4 | 4 | 4 | 4 | 4 |
| 5 | 5 | 5 | 5 | 5 | 4 | 4 | 4 | 3 |
| 3 | 4 | 3 | 3 | 3 | 2 | 4 | 4 | 4 |
| 5 | 4 | 5 | 4 | 4 | 3 | 4 | 2 | 2 |
| 2 | 4 | 5 | 4 | 5 | 4 | 5 | 4 | 4 |
| 2 | 5 | 5 | 5 | 5 | 4 | 5 | 4 | 4 |
| 2 | 2 | 2 | 3 | 2 | 2 | 2 | 2 | 2 |
| 3 | 3 | 2 | 4 | 2 | 2 | 2 | 3 | 3 |
| 2 | 2 | 2 | 4 | 1 | 2 | 2 | 2 | 4 |
| 5 | 3 | 4 | 3 | 4 | 3 | 3 | 3 | 3 |
| 3 | 3 | 3 | 2 | 2 | 3 | 3 | 3 | 3 |
| 4 | 5 | 4 | 2 | 2 | 4 | 4 | 4 | 4 |
| 2 | 4 | 3 | 4 | 4 | 4 | 3 | 4 | 4 |
| 5 | 5 | 4 | 4 | 4 | 5 | 5 | 5 | 4 |
| 4 | 5 | 4 | 5 | 4 | 4 | 5 | 5 | 5 |
| 4 | 4 | 5 | 5 | 5 | 5 | 5 | 5 | 4 |
| 4 | 4 | 5 | 4 | 4 | 4 | 5 | 4 | 5 |
| 4 | 4 | 4 | 4 | 4 | 4 | 3 | 4 | 3 |
| 4 | 5 | 4 | 4 | 4 | 4 | 4 | 4 | 4 |
| 3 | 4 | 3 | 4 | 3 | 3 | 4 | 4 | 3 |

| | | | | | | | | |
|---|---|---|---|---|---|---|---|---|
| 4 | 4 | 4 | 4 | 4 | 4 | 4 | 4 | 4 |
| 3 | 4 | 3 | 4 | 4 | 4 | 4 | 4 | 4 |
| 5 | 5 | 4 | 5 | 4 | 5 | 5 | 5 | 4 |
| 5 | 5 | 4 | 4 | 4 | 5 | 5 | 5 | 4 |
| 4 | 5 | 4 | 5 | 4 | 4 | 5 | 5 | 5 |
| 4 | 4 | 5 | 5 | 5 | 5 | 5 | 5 | 4 |
| 4 | 5 | 4 | 4 | 4 | 4 | 4 | 4 | 4 |
| 3 | 4 | 3 | 4 | 3 | 3 | 4 | 4 | 3 |
| 4 | 4 | 5 | 4 | 4 | 4 | 5 | 4 | 5 |
| 4 | 5 | 4 | 5 | 4 | 4 | 5 | 5 | 5 |
| 5 | 4 | 5 | 4 | 5 | 4 | 4 | 4 | 5 |
| 4 | 5 | 5 | 5 | 5 | 5 | 4 | 4 | 5 |
| 5 | 4 | 4 | 4 | 4 | 5 | 5 | 5 | 5 |
| 5 | 5 | 5 | 5 | 5 | 5 | 5 | 5 | 5 |
| 4 | 5 | 4 | 5 | 4 | 5 | 4 | 4 | 4 |
| 4 | 4 | 4 | 4 | 4 | 4 | 4 | 4 | 4 |
| 5 | 4 | 5 | 4 | 5 | 4 | 4 | 4 | 3 |
| 4 | 4 | 4 | 4 | 4 | 4 | 3 | 4 | 3 |
| 4 | 5 | 4 | 5 | 4 | 4 | 5 | 5 | 5 |
| 5 | 4 | 5 | 4 | 5 | 4 | 4 | 4 | 5 |
| 4 | 5 | 5 | 5 | 5 | 5 | 4 | 4 | 5 |
| 5 | 4 | 4 | 4 | 4 | 5 | 5 | 5 | 5 |
| 5 | 5 | 5 | 5 | 5 | 5 | 5 | 5 | 5 |
| 4 | 5 | 4 | 5 | 4 | 5 | 4 | 4 | 4 |
| 4 | 4 | 4 | 4 | 4 | 4 | 4 | 4 | 4 |
| 5 | 4 | 5 | 4 | 5 | 4 | 4 | 4 | 3 |
| 4 | 4 | 4 | 4 | 4 | 4 | 3 | 4 | 3 |
| 4 | 5 | 5 | 5 | 4 | 5 | 4 | 5 | 4 |
| 4 | 4 | 4 | 4 | 4 | 3 | 4 | 5 | 4 |
| 4 | 5 | 4 | 4 | 4 | 4 | 5 | 4 | 5 |
| 5 | 5 | 5 | 5 | 5 | 1 | 1 | 5 | 4 |
| 4 | 3 | 4 | 4 | 4 | 4 | 4 | 4 | 4 |
| 5 | 5 | 5 | 5 | 5 | 4 | 4 | 4 | 3 |
| 3 | 4 | 3 | 3 | 3 | 2 | 4 | 4 | 4 |
| 5 | 4 | 5 | 4 | 4 | 3 | 4 | 2 | 2 |
| 2 | 4 | 5 | 4 | 5 | 4 | 5 | 4 | 4 |
| 2 | 5 | 5 | 5 | 5 | 4 | 5 | 4 | 4 |
| 2 | 2 | 2 | 3 | 2 | 2 | 2 | 2 | 2 |
| 3 | 3 | 2 | 4 | 2 | 2 | 2 | 3 | 3 |
| 2 | 2 | 2 | 4 | 1 | 2 | 2 | 2 | 4 |
| 5 | 3 | 4 | 3 | 4 | 3 | 3 | 3 | 3 |
| 3 | 3 | 3 | 2 | 2 | 3 | 3 | 3 | 3 |
| 4 | 5 | 4 | 2 | 2 | 4 | 4 | 4 | 4 |
| 2 | 4 | 3 | 4 | 4 | 4 | 3 | 4 | 4 |
| 5 | 5 | 5 | 5 | 5 | 5 | 5 | 5 | 5 |
| 5 | 5 | 5 | 5 | 5 | 2 | 2 | 3 | 3 |
| 4 | 3 | 4 | 4 | 4 | 2 | 2 | 2 | 4 |

| | | | | | | | | |
|---|---|---|---|---|---|---|---|---|
| 5 | 5 | 5 | 5 | 5 | 3 | 3 | 3 | 3 |
| 3 | 4 | 3 | 3 | 3 | 3 | 3 | 3 | 3 |
| 5 | 4 | 5 | 4 | 4 | 4 | 4 | 4 | 4 |
| 2 | 4 | 5 | 4 | 5 | 4 | 3 | 4 | 4 |
| 2 | 5 | 5 | 5 | 5 | 5 | 5 | 5 | 5 |
| 2 | 2 | 2 | 3 | 2 | 4 | 5 | 4 | 4 |
| 4 | 4 | 5 | 5 | 5 | 1 | 1 | 5 | 4 |
| 4 | 4 | 5 | 4 | 4 | 4 | 4 | 4 | 4 |
| 4 | 5 | 4 | 5 | 5 | 4 | 4 | 4 | 3 |
| 5 | 4 | 5 | 3 | 3 | 2 | 4 | 4 | 4 |
| 4 | 5 | 5 | 4 | 4 | 3 | 4 | 2 | 2 |
| 5 | 4 | 4 | 4 | 5 | 4 | 5 | 4 | 4 |
| 5 | 5 | 5 | 5 | 5 | 4 | 5 | 4 | 4 |
| 4 | 5 | 4 | 3 | 2 | 2 | 2 | 2 | 2 |
| 4 | 4 | 4 | 4 | 2 | 2 | 2 | 3 | 3 |
| 4 | 5 | 4 | 2 | 2 | 5 | 5 | 5 | 5 |
| 2 | 4 | 3 | 4 | 4 | 5 | 5 | 4 | 4 |
| 5 | 5 | 5 | 5 | 5 | 5 | 4 | 5 | 4 |
| 5 | 5 | 5 | 5 | 5 | 5 | 5 | 4 | 4 |
| 4 | 3 | 4 | 4 | 4 | 4 | 5 | 4 | 5 |
| 5 | 5 | 5 | 5 | 5 | 5 | 5 | 4 | 5 |
| 3 | 4 | 3 | 3 | 3 | 5 | 5 | 5 | 5 |
| 5 | 4 | 5 | 4 | 4 | 5 | 5 | 5 | 5 |
| 5 | 5 | 4 | 5 | 4 | 5 | 5 | 5 | 5 |
| 4 | 5 | 4 | 4 | 5 | 4 | 5 | 5 | 4 |
| 4 | 5 | 4 | 5 | 5 | 5 | 5 | 5 | 5 |
| 4 | 4 | 5 | 4 | 4 | 5 | 5 | 4 | 5 |
| 5 | 4 | 5 | 5 | 5 | 5 | 4 | 4 | 4 |
| 4 | 5 | 4 | 5 | 4 | 4 | 4 | 4 | 4 |
| 4 | 4 | 5 | 4 | 4 | 5 | 5 | 5 | 4 |
| 4 | 5 | 5 | 4 | 5 | 4 | 5 | 4 | 5 |
| 5 | 4 | 4 | 4 | 4 | 5 | 5 | 5 | 5 |
| 5 | 5 | 5 | 5 | 5 | 5 | 5 | 5 | 5 |
| 5 | 5 | 4 | 5 | 4 | 5 | 5 | 4 | 4 |
| 4 | 4 | 4 | 4 | 4 | 4 | 4 | 4 | 4 |
| 5 | 4 | 5 | 4 | 5 | 4 | 4 | 4 | 4 |
| 4 | 4 | 4 | 4 | 4 | 4 | 3 | 4 | 3 |
| 4 | 5 | 4 | 5 | 4 | 4 | 4 | 4 | 4 |
| 5 | 5 | 5 | 4 | 5 | 5 | 4 | 5 | 4 |
| 4 | 4 | 5 | 5 | 5 | 5 | 5 | 5 | 5 |
| 5 | 4 | 4 | 5 | 5 | 4 | 5 | 5 | 5 |
| 4 | 5 | 4 | 5 | 4 | 5 | 4 | 5 | 4 |
| 5 | 5 | 4 | 5 | 4 | 4 | 5 | 5 | 4 |
| 5 | 4 | 4 | 5 | 4 | 4 | 4 | 4 | 4 |
| 4 | 4 | 5 | 5 | 4 | 4 | 4 | 4 | 5 |
| 4 | 4 | 5 | 5 | 4 | 4 | 5 | 5 | 4 |
| 4 | 4 | 5 | 4 | 5 | 5 | 5 | 5 | 5 |

| | | | | | | | | |
|---|---|---|---|---|---|---|---|---|
| 5 | 5 | 4 | 5 | 5 | 4 | 5 | 4 | 5 |
| 4 | 5 | 5 | 5 | 5 | 5 | 5 | 5 | 5 |
| 5 | 5 | 4 | 5 | 5 | 4 | 5 | 5 | 4 |
| 4 | 4 | 5 | 4 | 5 | 5 | 4 | 4 | 5 |
| 5 | 4 | 5 | 5 | 4 | 4 | 5 | 4 | 5 |
| 5 | 5 | 4 | 5 | 4 | 4 | 4 | 4 | 5 |
| 4 | 5 | 4 | 5 | 4 | 4 | 4 | 5 | 4 |
| 4 | 5 | 5 | 5 | 4 | 4 | 5 | 5 | 4 |
| 4 | 4 | 5 | 5 | 5 | 4 | 5 | 5 | 4 |
| 5 | 5 | 5 | 4 | 5 | 5 | 4 | 4 | 5 |
| 4 | 4 | 5 | 4 | 5 | 4 | 4 | 4 | 5 |
| 5 | 5 | 4 | 5 | 4 | 4 | 5 | 4 | 5 |
| 5 | 5 | 4 | 5 | 4 | 4 | 5 | 5 | 4 |
| 3 | 5 | 4 | 5 | 5 | 5 | 5 | 5 | 5 |
| 5 | 5 | 4 | 5 | 5 | 5 | 4 | 4 | 4 |
| 4 | 5 | 5 | 5 | 5 | 5 | 5 | 5 | 5 |
| 4 | 5 | 5 | 5 | 5 | 4 | 4 | 4 | 5 |
| 4 | 5 | 5 | 4 | 4 | 5 | 4 | 5 | 5 |
| 5 | 5 | 5 | 5 | 5 | 5 | 4 | 5 | 5 |
| 2 | 5 | 4 | 4 | 5 | 4 | 5 | 5 | 4 |
| 3 | 5 | 5 | 5 | 5 | 4 | 4 | 5 | 5 |
| 2 | 5 | 5 | 5 | 5 | 5 | 5 | 5 | 5 |
| 3 | 4 | 4 | 3 | 3 | 4 | 4 | 4 | 4 |
| 3 | 5 | 5 | 3 | 4 | 4 | 4 | 5 | 5 |
| 5 | 5 | 5 | 5 | 3 | 5 | 5 | 5 | 5 |
| 4 | 5 | 5 | 5 | 4 | 5 | 5 | 5 | 5 |
| 5 | 5 | 4 | 5 | 5 | 4 | 4 | 5 | 5 |
| 5 | 5 | 4 | 5 | 4 | 4 | 5 | 4 | 4 |
| 3 | 5 | 4 | 5 | 5 | 4 | 4 | 5 | 4 |
| 5 | 5 | 4 | 5 | 5 | 4 | 4 | 4 | 4 |
| 4 | 5 | 5 | 5 | 4 | 5 | 5 | 4 | 5 |
| 4 | 4 | 4 | 5 | 5 | 4 | 4 | 5 | 5 |
| 4 | 5 | 4 | 5 | 5 | 4 | 4 | 3 | 5 |
| 5 | 4 | 4 | 5 | 5 | 4 | 5 | 5 | 4 |
| 2 | 5 | 5 | 5 | 5 | 4 | 4 | 4 | 5 |
| 4 | 5 | 5 | 4 | 4 | 4 | 4 | 5 | 5 |
| 4 | 5 | 4 | 4 | 4 | 5 | 5 | 5 | 4 |
| 5 | 4 | 5 | 5 | 5 | 4 | 5 | 5 | 5 |
| 4 | 4 | 5 | 5 | 5 | 4 | 5 | 5 | 4 |
| 5 | 4 | 5 | 4 | 4 | 4 | 5 | 5 | 4 |
| 4 | 5 | 4 | 5 | 5 | 4 | 4 | 4 | 4 |
| 5 | 4 | 5 | 5 | 5 | 4 | 5 | 4 | 5 |
| 5 | 4 | 5 | 5 | 5 | 5 | 5 | 4 | 4 |
| 4 | 4 | 5 | 4 | 4 | 5 | 5 | 5 | 5 |
| 5 | 5 | 5 | 5 | 5 | 5 | 4 | 4 | 4 |
| 4 | 5 | 5 | 5 | 4 | 4 | 4 | 5 | 4 |
| 5 | 5 | 4 | 5 | 4 | 5 | 4 | 5 | 4 |

| | | | | | | | | |
|---|---|---|---|---|---|---|---|---|
| 5 | 5 | 4 | 5 | 5 | 4 | 5 | 5 | 4 |
| 3 | 5 | 5 | 4 | 5 | 5 | 5 | 4 | 5 |
| 5 | 4 | 5 | 4 | 4 | 5 | 4 | 4 | 5 |
| 4 | 5 | 4 | 4 | 5 | 5 | 5 | 4 | 5 |
| 4 | 5 | 4 | 5 | 4 | 4 | 5 | 5 | 4 |
| 5 | 4 | 5 | 4 | 5 | 5 | 5 | 5 | 4 |
| 5 | 4 | 5 | 5 | 4 | 4 | 5 | 5 | 4 |
| 5 | 4 | 5 | 5 | 4 | 5 | 4 | 5 | 5 |
| 4 | 4 | 5 | 4 | 5 | 5 | 4 | 5 | 4 |
| 4 | 5 | 4 | 5 | 4 | 5 | 4 | 4 | 5 |
| 5 | 5 | 4 | 5 | 4 | 4 | 4 | 4 | 5 |
| 4 | 5 | 4 | 5 | 4 | 4 | 5 | 5 | 4 |
| 4 | 5 | 5 | 4 | 5 | 5 | 4 | 5 | 5 |
| 4 | 5 | 5 | 5 | 5 | 5 | 4 | 4 | 4 |
| 4 | 4 | 5 | 4 | 4 | 4 | 5 | 4 | 5 |
| 4 | 4 | 5 | 5 | 5 | 5 | 5 | 5 | 4 |
| 4 | 4 | 5 | 4 | 4 | 4 | 5 | 4 | 5 |
| 4 | 5 | 4 | 5 | 4 | 4 | 5 | 5 | 4 |
| 5 | 4 | 4 | 4 | 4 | 5 | 5 | 5 | 5 |
| 5 | 4 | 4 | 4 | 4 | 5 | 5 | 5 | 3 |
| 5 | 4 | 4 | 4 | 4 | 5 | 5 | 5 | 5 |
| 5 | 5 | 5 | 5 | 5 | 5 | 5 | 5 | 5 |
| 5 | 5 | 5 | 5 | 4 | 4 | 4 | 4 | 3 |
| 5 | 4 | 4 | 4 | 4 | 5 | 5 | 5 | 5 |
| 4 | 5 | 4 | 5 | 4 | 4 | 5 | 5 | 5 |
| 5 | 5 | 5 | 5 | 4 | 4 | 4 | 4 | 5 |
| 5 | 4 | 4 | 4 | 4 | 5 | 5 | 5 | 5 |
| 5 | 4 | 4 | 4 | 4 | 5 | 5 | 5 | 5 |
| 4 | 5 | 4 | 5 | 4 | 4 | 5 | 5 | 5 |
| 5 | 4 | 4 | 4 | 4 | 5 | 5 | 5 | 5 |
| 5 | 5 | 5 | 5 | 4 | 4 | 4 | 4 | 5 |
| 4 | 4 | 5 | 4 | 4 | 4 | 5 | 4 | 5 |
| 4 | 5 | 4 | 5 | 4 | 4 | 5 | 5 | 5 |
| 4 | 5 | 4 | 5 | 4 | 4 | 5 | 5 | 5 |
| 4 | 4 | 5 | 4 | 4 | 4 | 5 | 4 | 5 |
| 4 | 4 | 4 | 4 | 4 | 4 | 3 | 4 | 3 |
| 4 | 5 | 4 | 4 | 4 | 4 | 4 | 4 | 4 |
| 3 | 4 | 3 | 4 | 3 | 3 | 4 | 4 | 3 |
| 4 | 4 | 4 | 4 | 4 | 4 | 4 | 4 | 4 |
| 3 | 4 | 3 | 4 | 4 | 4 | 4 | 4 | 4 |
| 5 | 5 | 4 | 5 | 4 | 5 | 5 | 5 | 4 |
| 5 | 5 | 5 | 5 | 5 | 1 | 1 | 5 | 4 |
| 4 | 3 | 4 | 4 | 4 | 4 | 4 | 4 | 4 |
| 5 | 5 | 5 | 5 | 5 | 4 | 4 | 4 | 3 |
| 3 | 4 | 3 | 3 | 3 | 2 | 4 | 4 | 4 |
| 5 | 4 | 5 | 4 | 4 | 3 | 4 | 2 | 2 |
| 2 | 4 | 5 | 4 | 5 | 4 | 5 | 4 | 4 |

| | | | | | | | | |
|---|---|---|---|---|---|---|---|---|
| 2 | 5 | 5 | 5 | 5 | 4 | 5 | 4 | 4 |
| 2 | 2 | 2 | 3 | 2 | 2 | 2 | 2 | 2 |
| 3 | 3 | 2 | 4 | 2 | 2 | 2 | 3 | 3 |
| 4 | 5 | 5 | 5 | 5 | 5 | 4 | 4 | 5 |
| 5 | 4 | 4 | 4 | 4 | 5 | 5 | 5 | 5 |
| 5 | 5 | 5 | 5 | 5 | 5 | 5 | 5 | 5 |
| 4 | 5 | 4 | 5 | 4 | 5 | 4 | 3 | 4 |
| 4 | 4 | 4 | 4 | 4 | 4 | 4 | 4 | 4 |
| 5 | 4 | 5 | 4 | 5 | 4 | 4 | 3 | 3 |
| 4 | 4 | 4 | 4 | 4 | 4 | 3 | 4 | 3 |
| 4 | 5 | 5 | 4 | 4 | 4 | 4 | 4 | 4 |
| 3 | 4 | 3 | 4 | 3 | 3 | 5 | 3 | 3 |
| 4 | 4 | 4 | 4 | 4 | 4 | 4 | 4 | 4 |
| 3 | 4 | 3 | 4 | 4 | 4 | 4 | 4 | 4 |
| 5 | 5 | 4 | 5 | 4 | 5 | 3 | 5 | 5 |
| 5 | 5 | 4 | 4 | 4 | 5 | 5 | 5 | 4 |
| 4 | 5 | 4 | 5 | 4 | 4 | 5 | 5 | 5 |
| 4 | 4 | 3 | 5 | 5 | 5 | 5 | 5 | 4 |
| 4 | 4 | 5 | 4 | 4 | 4 | 5 | 4 | 3 |

| CE1 | CE2 | CE3 | CE4 | ASD1 | ASD3 | ASD4 | ASD5 |
|---|---|---|---|---|---|---|---|
| 4 | 5 | 5 | 4 | 5 | 4 | 5 | 5 |
| 5 | 4 | 4 | 4 | 4 | 4 | 5 | 5 |
| 5 | 5 | 5 | 4 | 5 | 4 | 5 | 4 |
| 5 | 5 | 5 | 5 | 5 | 5 | 5 | 4 |
| 5 | 5 | 4 | 4 | 4 | 4 | 5 | 4 |
| 5 | 5 | 4 | 5 | 4 | 5 | 5 | 4 |
| 5 | 5 | 5 | 5 | 5 | 5 | 5 | 4 |
| 4 | 4 | 4 | 5 | 4 | 5 | 5 | 5 |
| 5 | 5 | 5 | 5 | 5 | 5 | 5 | 5 |
| 5 | 5 | 5 | 5 | 5 | 5 | 5 | 5 |
| 3 | 3 | 3 | 4 | 3 | 4 | 3 | 4 |
| 3 | 3 | 3 | 3 | 3 | 3 | 4 | 4 |
| 4 | 4 | 4 | 4 | 4 | 4 | 3 | 4 |
| 4 | 4 | 4 | 4 | 4 | 4 | 3 | 4 |
| 4 | 4 | 4 | 4 | 4 | 4 | 4 | 4 |
| 4 | 4 | 4 | 4 | 4 | 4 | 3 | 4 |
| 4 | 4 | 4 | 4 | 4 | 4 | 4 | 3 |
| 4 | 4 | 4 | 4 | 4 | 4 | 4 | 4 |
| 4 | 5 | 5 | 4 | 5 | 4 | 5 | 5 |
| 5 | 4 | 4 | 4 | 4 | 4 | 5 | 5 |
| 5 | 5 | 5 | 4 | 5 | 4 | 5 | 4 |
| 5 | 5 | 5 | 5 | 5 | 5 | 5 | 4 |
| 5 | 5 | 4 | 4 | 4 | 4 | 5 | 4 |
| 5 | 5 | 4 | 5 | 4 | 5 | 5 | 4 |
| 5 | 5 | 5 | 5 | 5 | 5 | 5 | 4 |
| 4 | 4 | 4 | 5 | 4 | 5 | 5 | 5 |
| 5 | 5 | 5 | 5 | 5 | 5 | 5 | 5 |
| 5 | 5 | 4 | 4 | 4 | 4 | 5 | 4 |
| 5 | 5 | 4 | 5 | 4 | 5 | 5 | 4 |
| 5 | 5 | 5 | 5 | 5 | 5 | 5 | 4 |
| 4 | 4 | 4 | 5 | 4 | 5 | 5 | 5 |
| 5 | 5 | 5 | 5 | 5 | 5 | 5 | 5 |
| 5 | 5 | 5 | 5 | 5 | 5 | 5 | 5 |
| 3 | 3 | 3 | 4 | 3 | 4 | 3 | 4 |
| 5 | 4 | 5 | 5 | 5 | 5 | 5 | 5 |
| 4 | 5 | 5 | 4 | 5 | 4 | 4 | 5 |
| 5 | 4 | 4 | 4 | 4 | 4 | 4 | 5 |
| 4 | 4 | 4 | 4 | 4 | 4 | 3 | 4 |
| 5 | 5 | 4 | 4 | 4 | 4 | 5 | 4 |
| 3 | 3 | 3 | 3 | 3 | 3 | 4 | 4 |
| 4 | 4 | 4 | 4 | 4 | 4 | 3 | 4 |
| 5 | 5 | 5 | 5 | 5 | 5 | 5 | 5 |
| 4 | 5 | 5 | 4 | 5 | 4 | 5 | 5 |
| 4 | 5 | 5 | 5 | 5 | 5 | 5 | 5 |
| 4 | 4 | 5 | 5 | 5 | 5 | 4 | 5 |
| 5 | 5 | 4 | 4 | 4 | 4 | 5 | 4 |

| | | | | | | | |
|---|---|---|---|---|---|---|---|
| 5 | 5 | 5 | 5 | 5 | 5 | 5 | 4 |
| 5 | 5 | 4 | 4 | 4 | 4 | 5 | 4 |
| 5 | 5 | 4 | 5 | 4 | 5 | 5 | 4 |
| 5 | 5 | 5 | 5 | 5 | 5 | 5 | 5 |
| 5 | 5 | 5 | 5 | 5 | 5 | 5 | 5 |
| 5 | 5 | 5 | 5 | 5 | 5 | 5 | 5 |
| 5 | 5 | 5 | 5 | 5 | 5 | 5 | 5 |
| 4 | 5 | 5 | 5 | 5 | 5 | 5 | 5 |
| 5 | 5 | 5 | 5 | 5 | 5 | 5 | 5 |
| 5 | 5 | 4 | 5 | 4 | 5 | 5 | 4 |
| 4 | 5 | 5 | 5 | 5 | 5 | 5 | 5 |
| 5 | 5 | 5 | 5 | 5 | 5 | 5 | 5 |
| 5 | 5 | 5 | 5 | 5 | 5 | 5 | 5 |
| 5 | 5 | 4 | 5 | 4 | 5 | 5 | 4 |
| 5 | 5 | 5 | 5 | 5 | 5 | 5 | 5 |
| 4 | 5 | 5 | 5 | 5 | 5 | 5 | 5 |
| 5 | 5 | 4 | 4 | 4 | 4 | 5 | 4 |
| 5 | 5 | 4 | 5 | 4 | 5 | 5 | 4 |
| 5 | 5 | 4 | 5 | 4 | 5 | 5 | 4 |
| 5 | 5 | 4 | 4 | 4 | 4 | 5 | 4 |
| 4 | 4 | 4 | 4 | 4 | 4 | 3 | 4 |
| 4 | 4 | 4 | 4 | 4 | 4 | 4 | 4 |
| 4 | 4 | 4 | 4 | 4 | 4 | 3 | 4 |
| 4 | 4 | 4 | 4 | 4 | 4 | 4 | 3 |
| 4 | 4 | 4 | 4 | 4 | 4 | 4 | 4 |
| 4 | 5 | 5 | 4 | 5 | 4 | 5 | 5 |
| 5 | 5 | 5 | 5 | 5 | 5 | 5 | 5 |
| 5 | 4 | 4 | 4 | 4 | 4 | 4 | 4 |
| 5 | 5 | 4 | 5 | 4 | 5 | 5 | 5 |
| 4 | 5 | 4 | 3 | 4 | 3 | 4 | 4 |
| 3 | 3 | 4 | 2 | 4 | 2 | 4 | 4 |
| 5 | 4 | 4 | 4 | 4 | 4 | 4 | 4 |
| 5 | 5 | 5 | 3 | 5 | 3 | 5 | 5 |
| 4 | 4 | 4 | 2 | 4 | 2 | 2 | 2 |
| 2 | 2 | 2 | 2 | 2 | 2 | 4 | 3 |
| 1 | 1 | 1 | 1 | 1 | 1 | 1 | 1 |
| 5 | 4 | 5 | 4 | 5 | 4 | 3 | 5 |
| 3 | 4 | 3 | 3 | 3 | 3 | 4 | 3 |
| 3 | 4 | 5 | 4 | 5 | 4 | 4 | 5 |
| 5 | 4 | 5 | 5 | 5 | 1 | 2 | 2 |
| 5 | 5 | 5 | 5 | 4 | 4 | 5 | 5 |
| 5 | 5 | 5 | 5 | 5 | 4 | 5 | 4 |
| 5 | 5 | 5 | 5 | 5 | 5 | 5 | 4 |
| 5 | 4 | 5 | 5 | 4 | 4 | 5 | 4 |
| 4 | 4 | 4 | 4 | 4 | 4 | 3 | 4 |
| 4 | 4 | 4 | 4 | 4 | 4 | 4 | 4 |
| 4 | 3 | 4 | 3 | 4 | 4 | 3 | 4 |

| | | | | | | | |
|---|---|---|---|---|---|---|---|
| 4 | 4 | 4 | 4 | 4 | 4 | 4 | 3 |
| 4 | 3 | 4 | 4 | 4 | 4 | 4 | 4 |
| 4 | 4 | 4 | 4 | 5 | 4 | 5 | 5 |
| 5 | 4 | 4 | 4 | 4 | 4 | 5 | 5 |
| 5 | 4 | 5 | 5 | 5 | 4 | 5 | 4 |
| 5 | 4 | 4 | 4 | 5 | 5 | 5 | 4 |
| 4 | 4 | 5 | 4 | 4 | 4 | 4 | 4 |
| 4 | 4 | 4 | 4 | 4 | 4 | 3 | 4 |
| 5 | 5 | 4 | 5 | 4 | 4 | 5 | 4 |
| 5 | 4 | 4 | 5 | 4 | 5 | 5 | 4 |
| 5 | 4 | 5 | 5 | 5 | 5 | 5 | 4 |
| 4 | 5 | 5 | 5 | 4 | 5 | 5 | 5 |
| 5 | 5 | 5 | 5 | 5 | 5 | 5 | 5 |
| 5 | 5 | 4 | 5 | 5 | 5 | 5 | 5 |
| 3 | 4 | 4 | 5 | 3 | 4 | 3 | 4 |
| 3 | 4 | 5 | 5 | 3 | 3 | 4 | 4 |
| 4 | 5 | 5 | 5 | 4 | 4 | 3 | 4 |
| 4 | 5 | 5 | 5 | 4 | 4 | 3 | 4 |
| 5 | 5 | 5 | 5 | 4 | 5 | 5 | 4 |
| 5 | 4 | 5 | 5 | 5 | 5 | 5 | 4 |
| 4 | 5 | 5 | 5 | 4 | 5 | 5 | 5 |
| 5 | 4 | 4 | 4 | 5 | 5 | 5 | 5 |
| 5 | 5 | 4 | 5 | 5 | 5 | 5 | 5 |
| 3 | 3 | 4 | 4 | 3 | 4 | 3 | 4 |
| 3 | 5 | 4 | 5 | 3 | 3 | 4 | 4 |
| 4 | 4 | 4 | 4 | 4 | 4 | 3 | 4 |
| 4 | 4 | 4 | 4 | 4 | 4 | 3 | 4 |
| 5 | 5 | 5 | 5 | 5 | 5 | 5 | 5 |
| 4 | 4 | 5 | 5 | 5 | 4 | 4 | 5 |
| 5 | 5 | 5 | 5 | 4 | 4 | 4 | 5 |
| 5 | 5 | 5 | 5 | 5 | 5 | 5 | 5 |
| 5 | 5 | 4 | 5 | 4 | 4 | 4 | 4 |
| 5 | 4 | 4 | 4 | 4 | 5 | 5 | 5 |
| 4 | 5 | 4 | 5 | 4 | 3 | 4 | 4 |
| 3 | 4 | 4 | 5 | 4 | 2 | 4 | 4 |
| 5 | 5 | 5 | 5 | 4 | 4 | 4 | 4 |
| 5 | 5 | 5 | 5 | 5 | 3 | 5 | 5 |
| 4 | 5 | 5 | 5 | 4 | 2 | 2 | 2 |
| 2 | 5 | 5 | 5 | 2 | 2 | 4 | 3 |
| 1 | 5 | 5 | 5 | 1 | 1 | 1 | 1 |
| 5 | 5 | 5 | 5 | 5 | 4 | 3 | 5 |
| 3 | 4 | 4 | 5 | 3 | 3 | 4 | 3 |
| 3 | 5 | 5 | 5 | 5 | 4 | 4 | 5 |
| 5 | 5 | 5 | 5 | 5 | 1 | 2 | 2 |
| 5 | 5 | 5 | 5 | 5 | 5 | 5 | 5 |
| 2 | 4 | 4 | 5 | 5 | 4 | 5 | 5 |
| 1 | 5 | 5 | 5 | 4 | 4 | 5 | 5 |

| | | | | | | | |
|---|---|---|---|---|---|---|---|
| 5 | 5 | 5 | 5 | 5 | 4 | 5 | 4 |
| 3 | 5 | 4 | 5 | 5 | 5 | 5 | 4 |
| 3 | 4 | 4 | 5 | 4 | 4 | 5 | 4 |
| 5 | 4 | 4 | 5 | 4 | 5 | 5 | 4 |
| 5 | 5 | 4 | 5 | 5 | 5 | 5 | 4 |
| 5 | 3 | 4 | 3 | 4 | 5 | 5 | 5 |
| 5 | 4 | 4 | 4 | 5 | 5 | 5 | 5 |
| 5 | 3 | 4 | 4 | 4 | 4 | 4 | 4 |
| 5 | 4 | 4 | 4 | 4 | 5 | 5 | 5 |
| 4 | 4 | 4 | 4 | 4 | 3 | 4 | 4 |
| 3 | 4 | 5 | 5 | 4 | 2 | 4 | 4 |
| 5 | 5 | 1 | 1 | 4 | 4 | 4 | 4 |
| 5 | 4 | 4 | 4 | 5 | 3 | 5 | 5 |
| 4 | 3 | 4 | 4 | 4 | 2 | 2 | 2 |
| 2 | 3 | 2 | 2 | 2 | 2 | 4 | 3 |
| 4 | 5 | 4 | 2 | 4 | 2 | 2 | 2 |
| 5 | 5 | 4 | 5 | 4 | 4 | 3 | 4 |
| 5 | 5 | 4 | 4 | 4 | 4 | 3 | 4 |
| 5 | 3 | 3 | 2 | 4 | 4 | 4 | 4 |
| 5 | 2 | 3 | 2 | 4 | 4 | 3 | 4 |
| 5 | 1 | 2 | 5 | 4 | 4 | 4 | 3 |
| 5 | 4 | 3 | 3 | 4 | 4 | 4 | 4 |
| 4 | 3 | 3 | 3 | 5 | 4 | 5 | 5 |
| 2 | 2 | 3 | 4 | 4 | 4 | 5 | 5 |
| 4 | 5 | 4 | 4 | 5 | 4 | 5 | 4 |
| 5 | 4 | 4 | 4 | 5 | 5 | 5 | 4 |
| 4 | 4 | 5 | 4 | 4 | 4 | 5 | 4 |
| 3 | 4 | 4 | 4 | 5 | 5 | 5 | 5 |
| 3 | 5 | 4 | 5 | 5 | 4 | 5 | 5 |
| 5 | 3 | 4 | 3 | 4 | 4 | 5 | 5 |
| 5 | 4 | 4 | 4 | 5 | 4 | 5 | 4 |
| 5 | 3 | 4 | 4 | 5 | 5 | 5 | 4 |
| 5 | 4 | 4 | 4 | 4 | 4 | 5 | 4 |
| 5 | 4 | 4 | 4 | 4 | 5 | 5 | 4 |
| 5 | 4 | 5 | 5 | 5 | 5 | 5 | 4 |
| 4 | 4 | 4 | 4 | 4 | 5 | 5 | 5 |
| 3 | 4 | 5 | 4 | 5 | 5 | 5 | 5 |
| 5 | 4 | 4 | 4 | 4 | 4 | 4 | 4 |
| 5 | 4 | 4 | 4 | 4 | 5 | 4 | 5 |
| 4 | 3 | 4 | 4 | 5 | 5 | 5 | 5 |
| 4 | 5 | 4 | 5 | 5 | 5 | 4 | 4 |
| 4 | 4 | 4 | 5 | 4 | 5 | 4 | 4 |
| 5 | 4 | 5 | 5 | 4 | 5 | 5 | 4 |
| 4 | 5 | 5 | 5 | 5 | 4 | 5 | 4 |
| 5 | 5 | 5 | 5 | 4 | 4 | 5 | 4 |
| 4 | 5 | 5 | 5 | 4 | 5 | 4 | 4 |
| 5 | 4 | 5 | 5 | 4 | 4 | 5 | 4 |

| | | | | | | | |
|---|---|---|---|---|---|---|---|
| 5 | 4 | 4 | 4 | 5 | 4 | 5 | 4 |
| 5 | 4 | 4 | 4 | 4 | 4 | 4 | 4 |
| 5 | 3 | 4 | 3 | 5 | 5 | 4 | 4 |
| 4 | 4 | 4 | 5 | 4 | 5 | 5 | 4 |
| 4 | 4 | 5 | 5 | 4 | 4 | 5 | 4 |
| 4 | 5 | 5 | 5 | 4 | 4 | 5 | 4 |
| 4 | 5 | 5 | 5 | 4 | 4 | 5 | 4 |
| 4 | 5 | 4 | 5 | 4 | 5 | 5 | 4 |
| 5 | 5 | 4 | 5 | 4 | 5 | 4 | 5 |
| 4 | 5 | 4 | 5 | 4 | 5 | 5 | 4 |
| 4 | 5 | 4 | 5 | 4 | 5 | 4 | 5 |
| 4 | 5 | 4 | 5 | 4 | 5 | 4 | 5 |
| 5 | 5 | 4 | 5 | 4 | 5 | 4 | 5 |
| 5 | 5 | 4 | 5 | 4 | 5 | 5 | 4 |
| 5 | 5 | 4 | 4 | 4 | 4 | 5 | 4 |
| 5 | 4 | 4 | 4 | 4 | 4 | 5 | 5 |
| 5 | 5 | 4 | 4 | 4 | 4 | 5 | 5 |
| 4 | 5 | 5 | 5 | 5 | 5 | 4 | 4 |
| 5 | 5 | 5 | 5 | 5 | 5 | 5 | 5 |
| 4 | 5 | 5 | 4 | 5 | 4 | 5 | 5 |
| 4 | 5 | 4 | 5 | 4 | 5 | 5 | 5 |
| 5 | 5 | 5 | 5 | 5 | 5 | 5 | 5 |
| 4 | 4 | 4 | 3 | 4 | 3 | 3 | 3 |
| 5 | 4 | 4 | 4 | 4 | 4 | 4 | 4 |
| 4 | 5 | 4 | 4 | 4 | 4 | 4 | 4 |
| 4 | 5 | 5 | 5 | 5 | 5 | 5 | 5 |
| 4 | 5 | 4 | 4 | 4 | 4 | 4 | 5 |
| 4 | 5 | 5 | 4 | 5 | 4 | 5 | 5 |
| 4 | 5 | 5 | 5 | 5 | 5 | 4 | 4 |
| 4 | 5 | 5 | 5 | 5 | 5 | 4 | 4 |
| 5 | 5 | 5 | 5 | 5 | 5 | 4 | 4 |
| 4 | 5 | 5 | 4 | 5 | 4 | 4 | 5 |
| 4 | 5 | 5 | 5 | 5 | 5 | 4 | 4 |
| 4 | 5 | 5 | 5 | 5 | 5 | 4 | 4 |
| 4 | 5 | 5 | 5 | 5 | 5 | 4 | 4 |
| 4 | 5 | 5 | 5 | 5 | 5 | 4 | 4 |
| 5 | 5 | 4 | 5 | 4 | 5 | 5 | 4 |
| 5 | 4 | 5 | 4 | 5 | 4 | 5 | 4 |
| 4 | 5 | 5 | 4 | 5 | 4 | 5 | 5 |
| 5 | 5 | 5 | 5 | 5 | 5 | 4 | 5 |
| 4 | 5 | 5 | 4 | 5 | 4 | 4 | 4 |
| 5 | 4 | 4 | 4 | 4 | 4 | 4 | 5 |
| 4 | 5 | 5 | 5 | 5 | 5 | 5 | 5 |
| 4 | 5 | 5 | 5 | 5 | 5 | 4 | 4 |
| 5 | 5 | 5 | 5 | 5 | 5 | 5 | 5 |
| 5 | 4 | 4 | 5 | 4 | 5 | 5 | 5 |
| 4 | 5 | 4 | 4 | 4 | 4 | 5 | 4 |

| | | | | | | | |
|---|---|---|---|---|---|---|---|
| 5 | 5 | 4 | 5 | 4 | 5 | 4 | 5 |
| 4 | 5 | 5 | 4 | 5 | 4 | 5 | 4 |
| 5 | 4 | 5 | 4 | 5 | 4 | 5 | 5 |
| 4 | 5 | 4 | 5 | 4 | 5 | 4 | 5 |
| 4 | 5 | 5 | 4 | 5 | 4 | 5 | 5 |
| 5 | 4 | 5 | 4 | 5 | 4 | 5 | 4 |
| 4 | 5 | 4 | 5 | 4 | 5 | 4 | 5 |
| 4 | 5 | 4 | 5 | 4 | 5 | 5 | 4 |
| 4 | 5 | 5 | 5 | 5 | 5 | 4 | 5 |
| 5 | 4 | 5 | 4 | 5 | 4 | 5 | 5 |
| 4 | 5 | 5 | 4 | 5 | 4 | 5 | 4 |
| 5 | 4 | 4 | 5 | 4 | 5 | 4 | 5 |
| 4 | 5 | 5 | 4 | 5 | 4 | 5 | 5 |
| 4 | 4 | 5 | 5 | 5 | 5 | 4 | 5 |
| 5 | 5 | 4 | 4 | 4 | 4 | 5 | 4 |
| 5 | 5 | 5 | 5 | 5 | 5 | 5 | 4 |
| 5 | 5 | 4 | 4 | 4 | 4 | 5 | 4 |
| 5 | 5 | 4 | 5 | 4 | 5 | 5 | 4 |
| 5 | 5 | 5 | 5 | 5 | 5 | 5 | 5 |
| 5 | 5 | 5 | 5 | 5 | 5 | 5 | 5 |
| 5 | 5 | 5 | 5 | 5 | 5 | 5 | 5 |
| 5 | 5 | 5 | 5 | 5 | 5 | 5 | 5 |
| 4 | 5 | 5 | 5 | 5 | 5 | 5 | 5 |
| 5 | 5 | 5 | 5 | 5 | 5 | 5 | 5 |
| 5 | 5 | 4 | 5 | 4 | 5 | 5 | 4 |
| 4 | 5 | 5 | 5 | 5 | 5 | 5 | 5 |
| 5 | 5 | 5 | 5 | 5 | 5 | 5 | 5 |
| 5 | 5 | 5 | 5 | 5 | 5 | 5 | 5 |
| 5 | 5 | 4 | 5 | 4 | 5 | 5 | 4 |
| 5 | 5 | 5 | 5 | 5 | 5 | 5 | 5 |
| 4 | 5 | 5 | 5 | 5 | 5 | 5 | 5 |
| 5 | 5 | 4 | 4 | 4 | 4 | 5 | 4 |
| 5 | 5 | 4 | 5 | 4 | 5 | 5 | 4 |
| 5 | 5 | 4 | 5 | 4 | 5 | 5 | 4 |
| 5 | 5 | 4 | 4 | 4 | 4 | 5 | 4 |
| 4 | 4 | 4 | 4 | 4 | 4 | 3 | 4 |
| 4 | 4 | 4 | 4 | 4 | 4 | 4 | 4 |
| 4 | 4 | 4 | 4 | 4 | 4 | 3 | 4 |
| 4 | 4 | 4 | 4 | 4 | 4 | 4 | 3 |
| 4 | 4 | 4 | 4 | 4 | 4 | 4 | 4 |
| 4 | 5 | 5 | 4 | 5 | 4 | 5 | 5 |
| 5 | 5 | 5 | 5 | 5 | 5 | 5 | 5 |
| 5 | 4 | 4 | 4 | 4 | 4 | 4 | 4 |
| 5 | 5 | 4 | 5 | 4 | 5 | 5 | 5 |
| 4 | 5 | 4 | 3 | 4 | 3 | 4 | 4 |
| 3 | 3 | 4 | 2 | 4 | 2 | 4 | 4 |
| 5 | 4 | 4 | 4 | 4 | 4 | 4 | 4 |

| | | | | | | | |
|---|---|---|---|---|---|---|---|
| 5 | 5 | 5 | 3 | 5 | 3 | 5 | 5 |
| 4 | 4 | 4 | 2 | 4 | 2 | 2 | 2 |
| 2 | 2 | 2 | 2 | 2 | 2 | 4 | 3 |
| 4 | 4 | 4 | 5 | 4 | 5 | 5 | 5 |
| 5 | 5 | 5 | 5 | 5 | 5 | 5 | 5 |
| 5 | 5 | 5 | 5 | 5 | 5 | 5 | 5 |
| 3 | 3 | 3 | 4 | 3 | 4 | 3 | 4 |
| 3 | 3 | 3 | 3 | 3 | 3 | 4 | 4 |
| 4 | 4 | 4 | 4 | 4 | 4 | 3 | 4 |
| 4 | 4 | 2 | 4 | 4 | 4 | 3 | 4 |
| 4 | 2 | 3 | 4 | 4 | 4 | 4 | 4 |
| 4 | 4 | 4 | 4 | 4 | 4 | 3 | 4 |
| 4 | 5 | 3 | 1 | 4 | 4 | 4 | 3 |
| 4 | 4 | 4 | 3 | 4 | 4 | 4 | 4 |
| 4 | 5 | 5 | 4 | 5 | 4 | 5 | 5 |
| 5 | 4 | 4 | 4 | 4 | 4 | 5 | 5 |
| 5 | 5 | 5 | 4 | 5 | 4 | 5 | 4 |
| 5 | 5 | 5 | 5 | 5 | 5 | 5 | 4 |
| 5 | 5 | 4 | 4 | 4 | 4 | 5 | 4 |
